# A Case of Conservative Surgery*Reported to Medical Club.

**Published:** 1880-07

**Authors:** J. W. Keene


### Clinical Reports.

---

### A CASE OF CONSERVATIVE SURGERY.*

REPORTED BY J. W. KEENE, M. D.

January 17, 1880, Jacob Spohn, aged 17, a healthy German boy, was caught by the forearm between cog-wheels in a barrel manufactory. He was brought to Dr. Briggs' office, where an

*Reported to Medical Club.

examination was made. He was sent to his home and visited immediately by Dr. B., who requested my assistance. The ulnar border and anterior aspect of the forearm was denuded of integument from the palm to the upper third; at the wrist there remained but an inch of skin over the radial border; the denuded space narrowed gradually upward; the skin hung in three strips one and a half inches wide over the border of the arm, with corresponding space between of the same width where the integument was entirely removed, thus showing the nature of the gearing in which the limb was caught; there were also two abrasions of the same width higher up, with similar spaces of intact skin separating them. On the palmar surface the wound extended an inch and a half into the palm, involving the entire width of the hand, dipping deep into the palmar tissues, and laying bare the flexor tendons over the wrist joint; the radius and radial artery were intact; the lower third of ulna was fractured in three places, and the laceration of the soft tissues rendered the fracture compound; the ulnar artery was severed, but the hemorrhage was not excessive; the temperature and sensation in the hand were good. On consultation it was decided to try to save the arm, although both physicians were doubtful of success. The patient and friends were informed that amputation might become necessary in a day or two, but that an attempt at preservation should be made.

Chloroform was administered; the lacerated tissues trimmed away; the lower third of the ulna was excised, and the proximal end of the ulnar artery found and ligated; the distal extremity eluded search. The anæsthetic was well borne. The arm was extended on a pillow covered with oil cloth, and tepid water dressing applied. Syrup of Dover's powder in dram doses was prescribed, and the patient was left in a comfortable condition.

Thirty-six hours later the arm was doing well, and was ordered dressed with tepid solution of carbolic acid—one dram in an ounce of glycerine to a pint of water.

Jan. 24. The arm was looking well. No slough of importance, but copious discharge of healthy pus from the entire granulating surface; cicatrization already established along the edges. There has been but slight constitutional disturbance and very little pain.

April 22. Patient was seen by me in Dr. Briggs' office. Wound all healed two weeks ago. Fingers moderately flexed, the little finger more than the others; it is also cold and has but little voluntary mobility. The other fingers have three-fourths of normal motion, the wrist joint one-third. Pronation is complete, supination fully three-fourths of normal. The defective motions are improving daily.

